# Perceptions and experiences of healthcare professionals on the guideline of management and treatment of anemia among children under 3 years of age during the COVID-19 pandemic

**DOI:** 10.17843/rpmesp.2022.391.9954

**Published:** 2022-03-31

**Authors:** Doris Delgado-Pérez, Juan Pablo Aparco, Sissy Espinoza-Bernardo, Margot Quintana-Salinas

**Affiliations:** 1 Instituto Centro de Investigación de Bioquímica y Nutrición, Universidad Nacional Mayor de San Marcos, Lima, Peru. Universidad Nacional Mayor de San Marcos Instituto Centro de Investigación de Bioquímica y Nutrición Universidad Nacional Mayor de San Marcos Lima Peru; 2 Centro Nacional de Alimentación y Nutrición, Instituto Nacional de Salud, Lima, Peru. Centro Nacional de Alimentación y Nutrición Instituto Nacional de Salud Lima Peru; 3 Departamento de Nutrición, Facultad de Medicina, Universidad Nacional Mayor de San Marcos, Lima, Peru. Universidad Nacional Mayor de San Marcos Departamento de Nutrición, Facultad de Medicina Universidad Nacional Mayor de San Marcos Lima Peru

**Keywords:** Anemia, Child, Qualitative Research, Perception, Health Care Personnel, COVID-19

## Abstract

**Objective.:**

To understand the perceptions and experiences of healthcare professionals on the application of the guideline for the management and treatment of anemia in children under 3 years old, during the COVID-19 pandemic, in metropolitan Lima, 2020.

**Materials and methods.:**

Phenomenological design. Individual semi-structured interviews were conducted with healthcare professionals: physicians, nurses and nutritionists working at the first level of care in the Ministry of Health in Lima, Peru. The interviews were conducted virtually with the participants, all of whom worked face-to-face in services providing care to children under 3 years old. Data analysis was thematic and NVivo software was used for coding.

**Results.:**

A total of 33 interviews with healthcare professionals were conducted between November 2020 and January 2021. Four themes emerged about the guideline: its feasibility, perceived imperfections, challenges in implementing it, and prospects for improvement. The health professionals interviewed perceived the guideline to be feasible to implement, but there were gaps in the indications that should have been more explicit. Nevertheless, they expressed their challenges and expectations for improvement.

**Conclusions.:**

Healthcare professionals perceived that it was feasible to use the guideline and emphasized their experiences overcoming perceived difficulties and weaknesses in the guidelines for anemia management and treatment.

## INTRODUCTION

Iron deficiency anemia has lifelong consequences, affecting health, education and work performance ^1^. Children under three years of age are the most vulnerable, as the prevalence of this deficiency remains high in different countries ^2^. Peru has had a stationary period between the years 2015-2019 with a prevalence above 40% ^3^ ^-^ ^6^.

The adverse effects of anemia on infant health have a significant cost for the family and society ^7^ ^,^ ^8^. To avoid these health, social and economic consequences, the World Health Organization (WHO) has recommended actions that include interventions to improve iron deficiency ^9^.

Many countries have implemented different strategies to address anemia; thus, the Peruvian government has implemented massive supplementation programs with various products such as ferrous sulfate, polymaltose iron, micronutrient powder (MNP), among others ^3^ ^-^ ^5^ ^,^ ^10^. Since the use of MNP, several studies have reported problems in the implementation of supplementation, especially in terms of adherence, distribution of micronutrients and difficulties in the process of receiving messages and supplements ^11^ ^-^ ^13^. One study reports that the role of health personnel in promoting the use and consumption of MNP is critical and that positive results are obtained with standardized messages and cultural adaptation to the local context ^14^.

On the other hand, the emergence of the COVID-19 pandemic significantly affected the provision of health services, including anemia care. Faced with the insecurity and fear of health workers and patients, the Ministry of Health (MINSA) issued Health Directive 099-2020 ^15^ for the management and control of anemia in the context of the pandemic health emergency. According to this new directive, physicians, nurses and nutritionists would be responsible for coordinating the appropriate measures to guarantee anemia prevention and control services ^15^. In addition, this directive also included new regulations, such as biosecurity measures to reduce contagion, and the use of technological means to solve the limitations of social isolation. However, these changes occurred so suddenly that it was not possible to assess whether health professionals were prepared to implement this new standard, and whether the health services had the conditions to guarantee biosecurity measures.

To date, in Peru, the mentioned standard is still in force and its effect on the management and control of anemia in the context of the COVID-19 pandemic has not been studied; however, it is known that standards and guidelines are useful to facilitate clinical practice. In addition, evidence shows that there are external, environmental and health professional factors that can affect their implementation ^16^; among them are time, resources, incentives, availability, costs, diagnostic tests, training, patients and cultural values ^17^.

Therefore, it is necessary to analyze the implementation of interventions against anemia, including the responses in the context of the pandemic and to try to understand how the new standard is being implemented, and to rescue the lessons learned in this process affected by fear, reduction of personnel and social isolation that configured a complex scenario for health care. The question that supports the study was: what is the perception and how has been the experience of the health professional regarding the implementation of the new standard for the management and treatment of anemia in children under three years of age?

Currently, the epidemiological scenario of COVID-19 is changing; however, the aforementioned standard is still in force. From this perspective, the aim of the study was to understand and assess the perceptions and experiences of the health professional on the implementation of the standard “Sanitary Directive 099-2020 for the management and treatment of anemia in children under three years of age in health facilities of the first level of care of MINSA”, during the COVID-19 pandemic, in Metropolitan Lima, in 2020. The findings of this study can serve as an input to design a new standard for the treatment and management of anemia that responds to the current health circumstances.

**KEY MESSAGES**

Motivation for the study: Anemia in children is highly prevalent in Peru and the COVID-19 pandemic is likely to further aggravate the problem. Directive 099-2020 was published to continue the management of anemia during the pandemic; however, health professional’s perception and experiences of its implementation are not known.

Main findings: Health professionals perceived and experienced that the norm in the emergency context was feasible for the continuity of anemia management. However, there were regulatory gaps that hindered its adequate implementation.

Implications: The perceived gaps and expectations for improvement could enrich future guidelines for the management and control of anemia in children under three years of age.

## MATERIALS AND METHODS

### Design

The study design was phenomenological ^18^, because it sought to understand the perceptions and experiences of the health professional during the processes involved in implementing anemia management and the control standard.

### Selection of participants

The study participants were health professionals, physicians, nurses and nutritionists who worked in health services of the first level of care of MINSA in Metropolitan Lima, and were in charge of the iron supplementation program for children under three years of age according to Health Directive 099-2020 ^15^. The eligibility criterion was that health professionals were working on-site in their service; 33 of 51 professionals invited agreed to participate voluntarily in the semi-structured in-depth interviews. Those not willing to participate stated that they did not have the time to do so.

The health professionals who participated were invited by telephone calls to collaborate with a virtual semi-structured interview. We used convenience and purposive sampling until information saturation was achieved ^19^. That is, no new important elements were found around the aim of the study and all the researchers considered that the number of interviews conducted provided sufficient information.

### Data collection

A semi-structured in-depth interview ^20^ was applied at the time scheduled with the professional. Health professionals worked in different services of the first level of mother-child care, such as medicine, nursing and nutrition; all used the guidelines on anemia control. The interviews were carried out between November 2020 and January 2021; the duration of each one was approximately 53 min. All interviews were conducted virtually using the Zoom.us implementation and conducted by a nutritionist with experience in qualitative data collection. It should be noted that the researchers did not work in any MINSA first level health facility and had no work relationship with the participants. All interviews were recorded to increase focus during the interview and facilitate subsequent analysis. A semi-structured guide was prepared containing open-ended questions on the following topics: feasibility of applying the standard, perception and experiences on the sufficiency of the standard, aspects less feasible to comply with and regulatory changes in the period 2017-2020. The semi-structured guide was intended to ensure that all topics were covered, and served as a template for the interview and was pre-tested with a population with the same characteristics.

### Data analysis

All recorded interviews were transcribed verbatim in Word format. The transcribed texts were subjected to thematic analysis ^21^. The first step of the analysis was to read all texts several times in order to have a general understanding of the data by all researchers. Next, the files were organized in NVivo software (QSR International, version Release 1.5.1-2020) ^22^ the coding process was quite complex because of the size of the questions and because of the researchers’ analysis. Coding was carried out by the first three authors (DDP, SE and JPA) independently, generating codes for each unit of analysis that were then compared between researchers. Initial codes were created according to a previously organized codebook as a basis for analysis. Significant categories of each theme were then searched for and an emerging codebook was created (supplementary material, Annex 1), which were grouped into sub-themes and themes. For the analysis process, we applied researcher triangulation ^23^. Afterwards, the findings were compared in several meetings for a better understanding of the data. In addition, the results were discussed, interpreted, and agreed upon among the four investigators to increase credibility. The level of consensus among different researchers of the same reality raises credibility, as well as the assurance that the level of congruence of the phenomena under study is strong and solid ^24^. We completed the 32-item COREQ evaluation checklist ^25^ (supplementary material, Annex 2).

### Ethical aspects

The study was approved by the Research Ethics Committee of the Faculty of Medicine of the Universidad Nacional Mayor de San Marcos (30-10-2020). The study was registered in the PRISA platform with registration EI00002115.

## RESULTS

**Figure 1:**
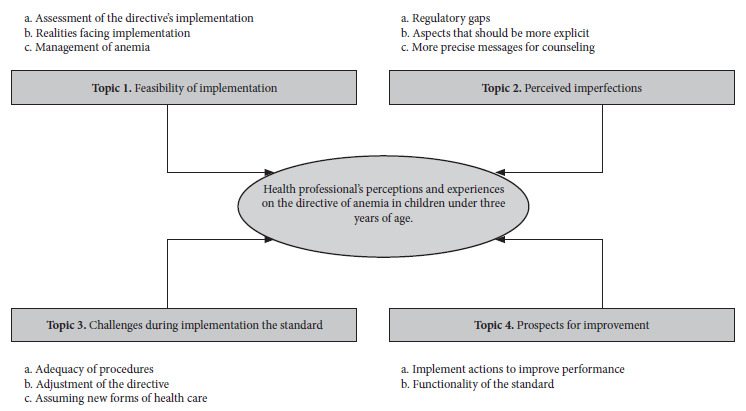
Topics and sub-topics on perceptions and experiences of health professionals on the directive of management and control of anemia in children under three years of age during the COVID-19 pandemic.

Thirty-three health professionals were interviewed, 4 physicians, 6 nurses and 23 nutritionists, with an average age of 39.8 (SD: 10.5) years. Eighty percent were female (supplementary material, annex 3). We identified 4 themes about the directive: feasibility of implementation, perceived imperfections, challenges in implementation, and prospects for improvement in the management and control of anemia (Figure 1).

### Topic 1. Feasibility of implementation

*Assessment of the directive’s implementation*

Twenty-six participants recognized the need to update the standard; in this regard, they mentioned that at the beginning of the pandemic there was much uncertainty about how to organize health care, although there were several proposals, there was no official one; this gap was partly filled with the standard “Health Directive No. 99”. Likewise, six of them stated that the directive facilitated work in the preventive management and treatment of anemia because it was specific and detailed, which allowed its implementation (Table 1). In addition, sixteen of them shared their experiences and, thanks to the standard that includes strategies such as virtual appointments, remote counseling and telemonitoring, they managed to continue with preventive supplementation and treatment (Table 1).

**Table 1:** Perceptions on the feasibility of applying the directive on management and control of anemia in children under three years of age.

Topics/categories	Quotes
Topic 1. Feasibility of applying the directive	
Assessment of the standard’s implementation	
Importance of updating the directive	*Umm, well, they always add a little more alternatives depending on the circumstances, for example, the last one (health directive 099) already talks about COVID and how we can manage (prevention and treatment of anemia); the previous one I think is a little more general, it talked about all age groups.* **Participant 12, nutritionist, age 32 **
Continuity of anemia management	*...There was no guidance on how to do this fundamental work in the country to combat anemia, which is deeply rooted in children under three years of age, and then this... helps us a lot to try to manage it from home, to manage it with remote counseling, telemonitoring....* **Participant 28, Physician, age 62 **
Realities facing implementation	
Complexity in the implementation of the new directive	*It was really hard work, we had to be in meetings, after meetings, soaking ourselves well in the norm, coordinating with those responsible for each area, because it was a coordination not only in my case with the doctor, but we also had to coordinate with the technician, with the nurse, with the obstetrician, so we had to coordinate in such a way that we did not expose the population and we did not expose ourselves....* **Participant 2, nutritionist, age 31 **
Scarce computer resources	*For example; I have had to have (sic) another cell phone to make calls because calling from my own cell phone..., sometimes at any time they would text me, Saturdays and Sundays, there was no time; that, for example, was a problem with COVID or anemia patients, they text you at any time of the day as if we were a call center.* **Participant 25 nutritionist, age 36 ** *There are still a lot of issues, because in order to carry out a good remote counseling and telemonitoring we also need digital tools that go hand in hand with this, so, yes, this strengthens us because it is a different world...* **Participant 26, nutritionist, age 31 **
Management of anemia	
Polymaltose iron shortage	*In itself, it is not the directive that changes, it is the annexes. For example, if you notice in the iron supplementation, it only mentions drops... it talks about polymaltose iron, ferrous sulfate and micronutrients, it is outdated; when currently there is not even polymaltose iron, there are no more drops of ferrous sulfate, and micronutrients have not been available for a long time...* **Participant 11, nutritionist, age 34 **
Management of adverse effects	*Well, I think the directive should be more specific in how it deals with the adverse effects of supplementation, iron, and what to do if the child does not continue with the supplementation.* **Participant 22, nurse, age 28 **
Indications of when supplementation is discontinued	*... Yes, it would be necessary for the rule to be explicit in the case of children who have interrupted their treatment for a long time or for more than 6 months, because many of them have not come since March, they have only arrived in July or September and 6 months have passed since the last visit or since... the last anemia treatment control.* **Participant 2, nutritionist, age 37 **
Hemoglobin measurement as a follow up	*The other point that is not specified is the hemoglobin dosage, because before we worked in the case of children with anemia, it was indicated at one month, at three months and at six months; but now it is at diagnosis, and from there at the end of the treatment, but the other regulations are pending and, in quotation marks, they are assumed, because if they see that the child is not exposed when it comes, but when it comes again for some vaccine, the detail comes that it was taken or not taken. For follow-up purposes, it has been taken, but the regulations do not define it as such.* **Participant 3, nutritionist, age 35 **

Source: Own elaboration

*Realities to face before implementation*

More than half of the participants expressed that they were emotionally unprepared to continue with their work due to the fear of COVID-19 infection and not knowing how to implement and comply with care in these circumstances, so they experienced uncertainty. In addition, they mentioned that they worked hard, first to learn about the new regulations and then to plan and implement the new strategies, especially in the flexibility of the supplement delivery time, a new patient flow, adapted environments, etc. Coordinated work and the willingness of the entire multidisciplinary team were necessary (Table 1).

The directive included modifications to standard care procedures, especially regarding the monitoring of virtual counseling. Many of them stated that they had experienced difficulties during the implementation, one of which was due to the absence and/or limited technological resources, such as not having a computer, or not having calling credit to make calls to patients. Twelve of them expressed their dissatisfaction and discomfort due to the lack of this basic equipment and had to use their personal telephones to make phone calls. This caused them to receive phone calls outside working hours and even on Saturdays and Sundays, sometimes for other types of care (Table 1).

*Management of anemia*

One of the complications of anemia treatment was the management of adverse effects. Most professionals stated that that polymaltose supplementation, which has fewer adverse effects, was in short supply. Twenty-one participants reported that the directive should include how to deal with adverse effects caused by iron supplementation (Table 1). Likewise, ten participants stated that there was difficulty when registering children with interrupted treatment and considered that the procedure for continuing iron supplementation when it is interrupted for more than six months should be specified. This situation occurred very frequently during the pandemic (Table 1).

### Topic 2. Perceived flaws

*Regulatory gaps*

The COVID-19 pandemic displaced the population, causing greater mobility and changes of domicile. This was related to the lack of continuous treatment of anemia. Participants expressed normative gaps, sixteen of them stated they encountered special situations when providing supplementation care. Some of the participants perceived that the directive did not include special conditions for supplementation, such as patients who previously did not come to the first level of care, but came due to the pandemic situation, for example: premature or very low birth weight infants (Table 2).

**Table 2:** Perceived gaps in the standard on management and control of anemia in children under three years of age

Topics/categories	Quotes
Topic 2. Perceived flaws in the directive	
Regulatory gaps	
Special dosing situations	*Well, it states that children with very low birth weight are focused more on maternal centers, but these patients are arriving in level 1-2, 1-3 facilities and in this case the medical staff has not handled it as it should be....* **Participant 3, nutritionist, age 35 **
Guidelines on what to do with recovered and continuing patients	*In recovered patients, uh...it doesn’t mention anything, maybe with those who finished their TA (discharge treatment), those who finished their treatment, they also don't mention the importance of that or how it is going to be managed, there is only one coding I have seen...well it doesn’t say it there either (in the directive), that is what...it doesn’t mention much about that, management of patients recovered from anemia.* **Participant 12, nutritionist, age 32**
Aspects that should be more specific	
Coverage of treatment distribution according to jurisdiction	*While it is true that the regulation says, both this and the previous one, it does not discriminate whether you have insurance or not, but...in this one it asks us, for example, to do it to all the children, it is done to all the children, but now for example we have children coming from different places and jurisdictions...who cannot be denied the hemoglobin dosage if they are in a range of children under 3 years of age. The directive tells you to give supplementation whether you have it or not, but the problem comes when you follow up....* **Participant 3, nutritionist, age 35**
Incorporate the nutritionist in supplement prescription.	*... in preventive activities in terms of health promotion, it intervenes in that aspect, but still... whether it is for 4 month old children, for 6 month old children without anemia or with anemia, the doctor gives it and puts it in the HIS (registry format) and everything, and really, for me and I do not know if this can be done from the school, but we have as much or the same capacity as a doctor I think to be able to indicate the supplement which is not a big deal, on the contrary I think we could cover a little more and it is an inconvenience I have always had.... as I repeat, the priority is...or the main axis, the one who gives the prescription is the professional nutritionist and it is not considered within the activity.* **Participant 4, nutritionist, age 33**
Increased accuracy of messages for counseling	
Advocate messages for counseling	*I believe that efforts should be made to increase the incidence of exclusive breastfeeding; I feel that this is a weak point that is not being given the necessary importance as a preventive activity for anemia.* **Participant 20, nutritionist, age 42 **
Indications on tools and supporting educational material	*...give some tips that are simpler for the professional to give to the community. A small, short healthy lifestyle promotion tip....* **Participant 28, physician, age 62**

Source: Own elaboration

Others stated that they did not know whether to provide full treatment or only for a few months. This presented inconveniences in the registry of recovered patients; thus, eight participants mentioned that the procedure for this registry was not clear (Table 2).

*Aspects that should be more specific*

Participants also mentioned that care coverage according to jurisdiction should be clearly described in the directive. This, with the aim of ensuring an orderly treatment provision and follow-up of patients. In this way, there would be no inconveniences in the measurement of indicators regarding the management and control of anemia (Table 2).

On the other hand, seven of the participants, especially the nutritionists, reported that their role is not properly defined in the directive, and that they should lead the process. Although nutritionists carry out preventive activities on anemia, they feel that they are limited regarding treatment, as they cannot prescribe the supplement or register their care activities (Table 2).

*More precise messages for counseling*

Twenty-one participants suggested that the term “personalized counseling” should be incorporated to reflect the activity that is actually being carried out. This consists of answering each mother’s questions and clarifying her doubts. It is also necessary to specify the duration and timing of this counseling in order to ensure that it is opportune (Table 2).

In addition, they recognized that the directive does not provide detailed messages or guidance on the different virtual activities. They emphasized the need for electronic educational material for dissemination with practical, short messages in simple language (Table 2).

### Topic 3. Challenges during implementation

*Adequacy of procedures*

The participants recounted their experiences during the mobility restrictions. Eleven of them stated that it was necessary to distribute tasks, in care and follow-up activities, among the few health personnel at their facility. Others mentioned that they strengthened the way they shared and delegated functions in order to achieve their objectives (Table 3). Twelve of them opted to optimize the patient flow for children with anemia in order to maintain order and comply with social distancing measures (Table 3).

**Table 3:** Challenges in applying the directive of management and control of anemia in children under three years of age.

Topics/categories	Quotes
Topic 3. Challenges in implementing the standard	
Adequacy of procedures	
Task distribution	*As there are staff who are still doing remote work, we coordinate with them so that they can call these, these mothers to come and we also work with goal 4, the health promoters, for example, they are given a list of 4-month-old children so they can visit and we have to work with them and see which children do not have the supplement....* **Participant 16, nutritionist, age 46 **
Improved patient flow	*Nutrition is in charge in this first instance of doing the dosage (there is a laboratory in the maternity center, licensed) if there is a laboratory, but in this case eh... we, both nursing and nutrition are doing the dosage directly in the consultation room (the dosage). We do have our equipment there, the hemoglobinometers; yes, we have those in each area, and they are performed there, and the result is seen there, and according to the result, well, it goes back to nursing for preventive care or to medicine.* **Participant 31, nurse, age 37**
Biosafety conditions for small environments	*So, the solution that has been given is that another different door has been made for the mothers who have children without risk and another door for the patients who have COVID another way, another route that does not cross. So, in that way we are protecting them. On the other hand, it has also been done as little walls, so I think there is no problem....* **Participant 30, nutritionist, age 65 **
“Adjustment” to the directive	
Adaptation of the prescription process	*...They gave us the authority to make visits, deliveries (of the supplement), and then the doctor would code (make the HIS registry), but I have already coordinated with the doctor so that I can write the prescription, fill it.... he even helps me with the HIS, filling it with his stamp...(other times) he (the doctor) stamps the prescription, but I already have it done, and that contributes more to the mother not having to wait so long, and it can be given to her quickly and avoid crowding.* **Participant 12, nutritionist, age 32 **
Assuming new forms of health care	
Changes in health care modality	*Yes, I agree with that part that it opens up a completely different world to the one we had lived in until before COVID, a completely different world of how to approach the mother, and now not necessarily having her in front of us physically, it makes the follow-up much easier, if we know how to handle it, I think....* **Participant 26, nutritionist, age 31 **
Disagreement with standardized dosage	*...In my opinion, the dose that has been given per age group is very low, this does not help us to strengthen the child’s ability to supplement as we used to do before according to weight. The supplementation should have been an average of what was being handled....* **Participant 14, nurse, age 35**

Source: Own elaboration

Others organized themselves for telephone follow-up of iron supplementation and home visits with adequate biosecurity equipment. Seven of them expressed their experiences in the conditioning of environments, in order to avoid infection by COVID-19 (Table 3).

*Adjustment of the directive*

The limited availability of physicians led to an “adjustment” or self-adaptation of supplementation care processes during social immobilization. Eleven of the participants reported that in some health facilities there was an internal agreement to facilitate the work of supplementation. That is, actions that deviated from this directive were carried out as part of the “adjustment” of the directive for its implementation in daily care. One of them was the delivery of the supplement by non-medical professionals with a medical prescription already prepared or to be regularized with the signature and stamp of the physician. All this with the purpose of not making the mother wait for a long time (Table 3). These practices caused some confusion at the beginning, because in an attempt to improve the distribution of supplements, they performed actions outside the norm.

*Assuming new forms of health care*

Twelve of the participants acknowledged that the changes stated in the directive regarding consultations or health care follow-up, forced them to adapt and overcome the barriers of social distance. They stated that previously they had great resistance to teleconsultation, telemonitoring and telephone follow-up. In this regard, they recognized that Information and Communication Technologies (ICT) are very important to take on these new challenges and that they require preparation, so they asked to be trained to facilitate their work and carry it out properly. Others took advantage of the use of applications known to them and contacted the mothers by telephone, which opened up more possibilities for guidance for some, while some feared that the mother would get tired of receiving continuous calls (Table 3).

Another practice they had to embrace from Health Directive 099-2020 was the standardized dose of iron supplements according to age. Sixteen health professionals agreed with this practice, because they recognized that, during the most critical context of the pandemic, it was not feasible to measure the child’s weight; especially because they would be exposed together with the mother to the contagion of COVID-19. However, eleven participants directly expressed their disagreement with the standardization of doses by weight (Table 3).

### Topic 4. Perspectives for improvement

*Implement actions to improve performance*

In the face of difficulties, the participants mentioned actions that could be improved in the directive, such as the weekly frequency of virtual follow-up. Twenty of the participants stated that telephone follow-up was difficult for different reasons. One of them was insufficient staff and they considered that more realistic goals should be established according to the possibilities of the health facilities (Table 4).

**Table 4:** Prospects for improving the standard on management and control of anemia in children under three years of age.

Topics/categories	Quotes
Topic 4. Prospects for improvement	
Implement actions to improve performance	
Honest follow-up	*...difficult to comply with, what is specific with respect to telephone follow-up, yes, it is a good support, but we could not do it alone, it would be clearer that the follow-up could be done by any technical or administrative personnel... That would be a strength, the indication of the follow-up.* **Participant 2, nutritionist, age 37 **
Clarify follow-up guidelines	*...I think that each one has guidelines to follow, how to carry them out and the commitments at the end, also how many commitments should be, because not all the information is going to be given over the phone, because it is not going to capture you, that is, I think it would be necessary to specify how this telephone follow-up should be....* **Participant 31, nurse, age 37**
Implement performance improvement actions	*...they tell us to carry out the remote counseling, but they do not give us some, I do not know, the, how to say it, the strategies to be able to carry out remote counseling, it is also an issue to be able to include these activities that we normally did, during consultation in the office we did not have to fill out anything and for us, even for the elderly it has been a little more tedious to be able to carry out remote counseling through the new platforms.* **Participant 27, nutritionist, age 31 **
Functionality of the directive	
Every child must undergo nutrition service	*...the issue of the nutrition part...the part of the nutrition consultation all of a sudden...because that is an important part, it doesn't say (the norm) that the child must go with nutrition, it doesn’t say anything about that, it only says that there are key messages (that they must receive) that he/she has to handle that, but the great majority of the centers have a nutritionist.* **Participant 12, nutritionist, age 32**
Exclusive service team	*... they ask us to serve everyone and... or another way, suddenly, if there is no money for an exclusive team, another strategy to serve... that there should be a nutritionist for children and a nutritionist for adults, try to make the flow shorter, try also that... or that there is an exclusive team to allow the nutritionist to fulfill his actual function as a nutritionist. And stop doing administrative functions.* **Participant 32, nutritionist, age 41**
Review vulnerability measurement instrument	One day, suddenly, I don’t know...that day something happened, the child got sick, suddenly he/she could not eat for some reason the three food groups, and suddenly, and it doesn’t mean that every day he/she eats like that, right? Suddenly it would be a more... or a more in-depth monitoring analysis, or to look at the week or the month, but not to look specifically at one day, which is the day before, which I don't think is accurate to consider someone vulnerable or classify them as vulnerable, right? **Participant 7, nutritionist, age 28**

Source: Own elaboration

Eleven participants considered that the mother should be committed to treatment compliance. They also stated that it should be specified how to supervise remote follow-up, especially when the health care center doesn’t have a virtual platform (Table 4).

Another aspect recommended by the participants was that MINSA should train all health personnel, including technicians, in the management and control of anemia. Training actions should include successful experiences in telemonitoring and teleconsultation of other facilities. On the other hand, they stated that the use of virtual platforms was very tedious, and considered that there should be more virtual counseling (Table 4).

*Functionality of the directive*

Participants stated that the directive should be more functional (more practical and useful); some of the recommendations included having an automated system, and expanding indications of hemoglobin measurement. Twelve participants mentioned that it should be specified that every attended child should go through the nutrition service, as long as the facility has a nutritionist. In addition, there should be an exclusive anemia care team (Table 4).

## DISCUSSION

This study provides an overview of the health professional’s perceptions and experiences regarding the current directive for the management and treatment of anemia in children under three years of age during the COVID-19 pandemic in the year 2020. In this sense, we found that health professionals perceived that the directive was useful and feasible to apply; in addition, they shared their experiences when providing anemia treatment and follow-up with few resources and new care regulations such as the use of virtual media. However, many encountered challenges during implementation and, in some cases, adapted procedures for the care of mothers and children. Likewise, the responsibility of caring for children in an emergency context, the fear of contagion and the sudden changes were challenges assumed by health professionals and, within the framework of this experience, they expressed their perspectives about improving the regulations to comply with the management of anemia.

Based on our results, several of the health professionals expressed the feasibility of applying the directive in the context of the COVID-19 pandemic. Some considered that it was an update of the previous directive or an annex, but that it contributed to the return to activities regarding recovery and prevention of anemia. According to the statements made by doctors, nurses and nutritionists, one of the most important aspects was that the directive included virtual care such as remote counseling and telephone follow-up. This allowed them to overcome distance and continue with supplementation. A study in health professionals found that telephone communication facilitated patient follow-up, especially for managing appointments ^26^, similar to what was found in our study, although there was an overload of staff activities, especially for patient follow-up. In addition, previous research shows that communication between the service provider and the patient improves treatment success and adherence to treatment ^27^. On the other hand, the implemented strategies, such as task distribution and improvement of the patient flow, made it possible to adapt to the context and improve care.

National guidelines are designed for the treatment of diseases in several countries, a particular case is anemia in children under three years of age. However, there are several challenges involved in their implementation. In our study, like that of Helseth *et al*. ^28^, health personnel were assigned new tasks and responsibilities with the new guideline, but they felt that they were not sufficiently prepared, nor were they supported by additional resources. Health professionals identified challenges in implementing the guidelines at several levels: at the health system level (health facility resources); health personnel level (perceived competence, burden of responsibility, attitudes); treatment level (supplementation dosage and monitoring instruments); and professional level (skepticism towards standardized iron supplementation dosage). All this had an impact on the low expectations of health professionals regarding the standard. Other studies show, in addition to the aforementioned drawbacks, the proliferation of guidelines, the redundancy of the topics addressed and biases in their methodology ^29^.

Due to the urgent implementation of the regulations in the context of the pandemic, many procedures had to be adapted. Although a guideline is a priority for decision making in health care, its acceptability and use by the health professional is complex and is influenced by several factors such as profession, knowledge, perceptions and attitudes. According to Solá *et al*, the use of guidelines in daily practice involves a process of implementation, which includes dissemination and training in these documents, facilitating elements that intervene in a process of positive feedback ^29^.

This study shows activities and procedures that have been adjusted to comply with the directive, showing that there are aspects to be reviewed. In the absence of physicians, several of the interviewed professionals (nurses and nutritionists) had to opt for providing the supplement and then complete the prescription procedure. In addition, they had to distribute tasks for the management of anemia among the available professionals, improve the face-to-face patient flow and changes in the modality of providing health care consultations and follow-up. This shows that the current directive lacks precision and needs to be adapted to the reality of each facility and region, in addition to having mechanisms to regulate them. The identification of barriers to the implementation and compliance with the guidelines should be analyzed in advance so that the designed strategies can be adapted to each specific context.

The COVID-19 pandemic swept the world, interrupting all human activities, mainly due to the fear of SARS-CoV-2 infection. In this context, treatment for anemia dropped sharply. Faced with this reality, the Health Directive 099-2020 emerged as an adequate response to guarantee the continuity of actions against anemia ^15^; however, the speed with which COVID-19 spread, the great economic, social, sanitary, psychological changes and the new situations in health care made it difficult for a norm to be sufficient to address the complex and dynamic scenario of the pandemic.

This inadequacy of the directive was perceived by the health professional who revealed gaps, emphasizing that no guidelines were included for the care of patients who did not attend first level facilities such as premature children, those with low birth weight, in addition to migrant children coming from high altitude areas; they also mentioned that there are no guidelines for continuous follow-up of children in mobility conditions (migrants). These gaps in directives during the pandemic have also been reported in other studies such as that of Shahil Feroz and Pradhan in Pakistan, where health personnel had to provide medical care without clear procedures ^30^, and the study by Iyer *et al*. in India, who recommends the development of practice guidelines (in this case surgical) appropriate to the pandemic, as well as frequent updating ^31^.

Another gap in this directive is that it does not refer to the means and resources needed to develop telehealth care; the participants mentioned the lack of resources, from basic aspects such as the financing of telephone calls, to the need for a virtual platform and digital material to develop remote counseling and telemonitoring. In this regard, the evidence shows a significant impact of telehealth for the prevention, diagnosis and treatment of some diseases; however, the implementation of telehealth implies some minimum requirements for both the health provider and the patient, and when these are not guaranteed, they constitute barriers to access to health services ^32^.

One of the limitations of the study was that data collection was done by video calls through the Zoom platform, which could restrict interaction; however, given the context, other studies have also used this strategy and provided recommendations, which we considered during the study to optimize the process ^33^. Other limitations were that only one data collection technique was applied (semi-structured in-depth interview); however, applying the interview in a direct and open manner was sufficient to saturate the information; and that the data were not returned to the participants for corroboration. The strengths of the study were that in order to guarantee the credibility of the data, the study applied two types of triangulation: triangulation by researchers and triangulation of data sources, considering the points of view of health professionals involved with anemia: physicians, nurses and nutritionists; in addition, another strength was that the sample was made up of health professionals from all the health directorates (DIRIS) of Metropolitan Lima and Callao.

In conclusion, our findings show that health professionals perceived that the directive contributed to continue with the management of anemia in a pandemic context, and the experiences showed the challenges during their implementation, which they tried to solve by modifying some indications according to their reality in order to ensure that children continue with the treatment and control of anemia. It is convenient to take into account the positive aspects expressed by the participants, as well as to consider the aspects that need to be clarified and added to future norms to make them more explicit and useful in the fight against anemia.
