# Supplementary material for: Perceptions and experiences of healthcare professionals on the guideline of management and treatment of anemia among children under 3 years of age during the COVID-19 pandemic
Source: Rev Peru Med Exp Salud Publica. 2022 Mar 31;39(1):24–35. doi: 10.17843/rpmesp.2022.391.9954 (PMC11397691; doi:10.17843/rpmesp.2022.391.9954)
Supplement: Supplementary material. — Available in the electronic version of the RPMESP. [file rpmesp-39-01-9954-s001.pdf]

MATERIAL SUPLEMENTARIO

Anexo 1. Árbol de códigos iniciales

Carpeta	Códigos	Descripción	Archivos	Referencias
Códigos\\thematic framework	T1 Factibilidad	Experiencias de implementar la estrategia anemia<br>Continuar con manejo<br>Complejidad implementación	33	255
Códigos\\thematic framework	T2 Imprecisiones de la norma	Desacuerdo dosificación estandarizada<br>Es suficiente la norma para control y manejo de la anemia. Vacíos normativos	33	137
Códigos\\thematic framework	T3 Desafíos aplicar la norma	Frente a las nuevas prácticas cuáles fueron los desafíos de aplicación de la norma: adaptaciones, dosis, procedimientos, ambientes	33	165
Códigos\\thematic framework	T4 Perspectiva de mejora	Recomendaciones sobre pautas de seguimiento, funcionalidad de la norma, acciones para mejorar el desempeño. Logros alcanzados. Rol de los profesionales. Prioridades para el trabajo virtual.	33	125

Fuente: elaboración propia

Anexo 2. Lista de chequeo *Consolidated criteria for reporting qualitative studies (COREQ)": 32-item* [25]

Ítem	Guide Questions/Description	Página- estudio
**Domain 1: Research team and reflexivity**		
1. Interviewer/facilitator	Which author/s conducted the interview or focus group?	Página 6 nutricionista entrenado
2. Credentials	What were the researcher´s credentials? (e.g., PhD, MD)	Página 1 - DD–MQ PhD
JPA – SE – M Sc.		
3. Occupation	What was their occupation at the time of the study?	Página 6 - DD-JPA-SE-MQ investigadores
4. Gender	Was the researcher male or female?	Página 1 - tres mujeres y un hombre
5. Experience and training	What experience or training did the researcher have?	Página 6 -DD-JPA-SE-MQ investigadores con experiencia previa en estudios cualitativos
Relationship with participants		
6. Relationship established	Was a relationship established prior to study commencement?	Página 7 – no hubo relación
7. Participant knowledge of the interviewer	What did the participants know about the researcher? (e.g., personal goals, reasons for doing the research).	Página 7 – se les proporcionó información antes de participar en el estudio (consentimiento informado)
8. Interviewer characteristics	What characteristics were reported about the interviewer/facilitator? (e.g., bias, assumptions, reasons and interests in the research topic).	Página 7 – se explicó las características de la entrevista.
**Domain 2: Study design**		
Theoretical framework		
9. Methodological orientation and Theory	What methodological orientation was stated to underpin the study? e.g., grounded theory, discourse analysis, ethnography, phenomenology, content analysis	Página 6 y 7 - diseño fenomenológico, análisis temático.
Participant selection		
10. Sampling	How were participants selected? e.g., purposive, convenience, consecutive, snowball	Página 6
11. Method of approach	How were participants approached? (e.g., face-to-face, telephone, mail, email).	Página 6
12. Sample size	How many participants were in the study?	Página 6
13. Non-participation	How many people refused to participate or dropped out? Reasons?	Página 6
Setting		
14. Setting of data collection	Where was the data collected? e.g., home, clinic, workplace	Página 6 – virtual Plataforma Zoom
15. Presence of non- participants	Was anyone else present besides the participants and researchers?	Página 6 – todas fueron entrevistas individuales.
16. Description of sample	What are the important characteristics of the sample? (e.g., demographic data, date).	Página 6 - sección Resultados.
Data collection		
17. Interview guide	Were questions, prompts, guides provided by the authors? Was it pilot tested?	Página 7
18. Repeat interviews	Were repeat inter views carried out? If yes, how many?	No. Descritas en la página 7.
19. Audio/visual recording	Did the research use audio or visual recording to collect the data?	Página 6
20. Field notes	Were field notes made during and/or after the interview or focus group?	No, estas fueron grabadas. Página 6
21. Duration	What was the duration of the interviews or focus group?	Página 6
22. Data saturation	Was data saturation discussed?	Página 6
23. Transcripts returned	Were transcripts returned to participants for comment and/or correction?	No
**Domain 3: analysis and findings**		
Data Analysis		
24. Number of data coders	How many data coders coded the data?	Página 7
25. Description of the coding tree	Did authors provide a description of the coding tree?	Página 7
26. Derivation of themes	Were themes identified in advance or derived from the data?	Página 7
27. Software	What software, if applicable, was used to manage the data?	Página 7
28. Participant checking	Did participants provide feedback on the findings?	No
Reporting		
29. Quotations presented	Were participant quotations presented to illustrate the themes/findings? Was each quotation identified? (e.g., participant number).	Página 7 a 12 – figura 1.
30. Data and findings consistent	Was there consistency between the data presented and the findings?	Si fue consistente. Página 7 a 12.
31. Clarity of major themes	Were major themes clearly presented in the findings?	Si fue consistente. Página 7 a 12. – tabla 1 a 4.
32. Clarity of minor themes	Is there a description of diverse cases or discussion of minor themes?	En la sección de Discusión se presenta los principales tema discutidos, Fig. 1 se resume los temas encontrados con las principales subcategorías.

Fuente: Tong A, Sainsbury P, Craig J. Consolidated criteria for reporting qualitative research (COREQ): a 32-item checklist for interviews and focus groups. International Journal for Quality in Health Care. 2007. Volume 19, Number 6: pp. 349–357

Anexo 3. Características de los participantes (n=33)

**Característica**	**n**	**%**
Edad, años, promedio (DS[a])	39,85	10,55
Tiempo de trabajo en el CS[b], años, mediana (Rango)	5,00	(30 - 0,4)
Sexo		
Femenino	29	88
Masculino	4	12
Profesión		
Enfermeras	6	18
Médicos	4	12
Nutricionistas	23	70
Tipo de servicio CS[b]		
Tópico de enfermería	2	4
Servicio de CRED[c]	4	12
Medicina general	4	12
Nutrición	23	70

Fuente: elaboración propia
[a] DS= desviación estándar
[b] CS= establecimiento de salud, primer nivel de atención
[c] CRED= servicio de crecimiento y desarrollo
