# Supplementary material for: Perceptions and experiences of healthcare professionals on the guideline of management and treatment of anemia among children under 3 years of age during the COVID-19 pandemic
Source: Rev Peru Med Exp Salud Publica. 2022 Mar 31;39(1):24–35. doi: 10.17843/rpmesp.2022.391.9954 (PMC11397691; doi:10.17843/rpmesp.2022.391.9954)
Supplement: Translation

# Translation: Percepciones y experiencias de los profesionales de la salud sobre la aplicación de la norma de manejo y tratamiento de la anemia en menores de tres años durante la pandemia de la COVID-19

**Authors:** Doris Delgado-Pérez, Juan Pablo Aparco, Sissy Espinoza-Bernardo, Margot Quintana-Salinas

**Affiliations:** 1 Instituto Centro de Investigación de Bioquímica y Nutrición, Universidad Nacional Mayor de San Marcos, Lima, Perú.; 2 Centro Nacional de Alimentación y Nutrición, Instituto Nacional de Salud, Lima, Perú.; 3 Departamento de Nutrición, Facultad de Medicina, Universidad Nacional Mayor de San Marcos, Lima, Perú.

**Keywords:** Anemia, Niño, Investigación Cualitativa, Percepción, Personal de Salud, COVID-19

## Abstract

**Objetivos.:**

Comprender las percepciones y experiencias de los profesionales de la salud sobre la aplicación de la directiva para el manejo y tratamiento de anemia en niños menores de tres años, durante la pandemia de la COVID-19 en Lima Metropolitana, año 2020.

**Materiales y métodos.:**

Estudio de diseño fenomenológico. Se realizaron entrevistas semiestructuradas individuales a profesionales de salud: médicos, enfermeras y nutricionistas que trabajaban en el primer nivel de atención en el Ministerio de Salud de Lima, Perú. Las entrevistas se llevaron a cabo en forma virtual. Todos laboraban en forma presencial en servicios de atención a niños menores de tres años. El análisis de datos fue temático, para las codificaciones se utilizó el software NVivo.

**Resultados.:**

Se realizaron 33 entrevistas a profesionales de la salud, entre noviembre de 2020 a enero de 2021. Surgieron cuatro temas emergentes sobre la norma: su factibilidad, imperfecciones percibidas, los desafíos para implementarla y las perspectivas de mejora. Los profesionales entrevistados percibieron que la norma era factible de ejecutarla, pero presentaba vacíos en las indicaciones que deberían estar más explícitos los aspectos del manejo y tratamiento de la anemia. A pesar de ello dieron a conocer sus desafíos y expectativas de mejora.

**Conclusiones.:**

Los encuestados percibieron que fue factible utilizar la norma y destacaron sus experiencias para lograr superar las dificultades pese a encontrar vacíos en la normativa para el manejo y tratamiento de la anemia.

## INTRODUCCIÓN

La anemia por deficiencia de hierro tiene consecuencias durante toda la vida, afectando la salud, la educación y el rendimiento laboral^ (^ ^1^. Los más vulnerables son los menores de tres años, pues la prevalencia de esta deficiencia sigue siendo alta en diferentes países^ (^ ^2^. El Perú ha tenido un periodo estacionario entre los años 2015-2019 con una prevalencia por encima del 40% ^3^ ^-^ ^6^.

Los efectos adversos de la anemia en la salud del infante se traducen en costos para la familia y la sociedad ^7^ ^,^ ^8^. Para evitar estas pérdidas sanitarias, sociales y económicas la Organización Mundial de la Salud (OMS) ha recomendado acciones que incluyan intervenciones que mejoren el estado de hierro ^9^. 

Muchos países han implementado diferentes estrategias frente a la anemia; así, el Estado Peruano ha ejecutado programas masivos de suplementación con diversos productos como sulfato ferroso, hierro polimaltosado, micronutrientes en polvo (MNP), entre otros ^3^ ^-^ ^5^ ^,^ ^10^. A partir del uso de MNP, diversos estudios reportaron problemas de implementación en la suplementación sobre todo en adherencia, distribución de micronutrientes y dificultades en el proceso de recepción de mensajes y suplementos ^11^ ^-^ ^13^. Un estudio reporta que el rol del personal de salud para promover el uso y consumo de MNP es crítico y que se obtienen resultados positivos con mensajes estandarizados y con adecuación cultural al contexto local ^14^. 

Por otra parte, la aparición de la pandemia de la COVID-19 afectó notablemente la prestación de servicios de salud, incluyendo las atenciones de anemia. Frente a la inseguridad y temor de los trabajadores de salud y los pacientes, el Ministerio de Salud (MINSA) elaboró la Directiva Sanitaria 099-2020 ^15^ para el manejo y control de la anemia en el contexto de emergencia sanitaria por la pandemia. Según esta nueva directiva el médico, la enfermera y la nutricionista serían los responsables de hacer las coordinaciones pertinentes para garantizar las prestaciones de prevención y control de la anemia ^15^. Además, estas disposiciones también incluían una serie de nuevas medidas, como las de bioseguridad para reducir el contagio, y el uso de medios tecnológicos para solucionar las limitaciones del aislamiento social. Sin embargo, estos cambios se produjeron tan repentinamente que no se pudo evaluar si los profesionales de la salud estaban preparados para implementar esta nueva norma, y si los servicios de salud contaban con las condiciones para garantizar las medidas de bioseguridad.

A la fecha, en el Perú, la referida norma sigue vigente y no se ha estudiado su efecto en el manejo y control de la anemia en el contexto de pandemia de la COVID-19; sin embargo, se conoce que las normas y guías son útiles para facilitar la práctica clínica. Además, la evidencia muestra que existen factores externos, ambientales y profesionales de salud que pueden afectar su implementación ^16^; entre ellos están el tiempo, recursos, incentivos, disponibilidad, costos, pruebas de diagnóstico, capacitación; los pacientes y los valores culturales ^17^.

Por ello, es necesario investigar la implementación de las intervenciones contra la anemia, incluyendo las respuestas en el contexto de la pandemia y tratar de comprender cómo se está ejecutando la nueva norma, y rescatar las lecciones aprendidas en este proceso afectado por el temor, reducción de personal y aislamiento social que configuraron un escenario complejo para las atenciones en salud. La interrogante que sustenta el estudio fue ¿cómo se percibe y cómo ha sido la experiencia del profesional de salud respecto a la aplicación de la nueva norma para el manejo y tratamiento de anemia en niños menores de tres años?.

Actualmente, el escenario epidemiológico de la COVID-19 está cambiando; sin embargo, la norma citada sigue vigente. Desde esa perspectiva, el objetivo del estudio fue comprender y valorar las percepciones y experiencias del profesional de salud sobre la aplicación de la norma «Directiva Sanitaria 099-2020 para el manejo y tratamiento de anemia en niños menores de tres años de establecimientos de salud del primer nivel de atención del MINSA», durante la pandemia de la COVID-19, en Lima Metropolitana, en el 2020. Los hallazgos del presente estudio pueden servir de insumo para elaborar una nueva norma de tratamiento y manejo de la anemia que responda a las circunstancias sanitarias actuales.

**MENSAJES CLAVE**

Motivación para realizar el estudio: el Perú tiene prevalencias elevadas de anemia infantil y la pandemia por la COVID-19 probablemente agrave más el problema. Se publicó la Directiva 099-2020 para continuar el manejo de la anemia durante la pandemia; sin embargo, no se conoce la percepción y experiencias del profesional de salud, sobre su aplicación.

Principales hallazgos: los profesionales de la salud percibieron y experimentaron que la norma en el contexto de emergencia fue factible para la continuidad del manejo de la anemia. Sin embargo, se presentaron vacíos normativos que obstaculizaron su adecuada implementación.

Implicancias: los vacíos percibidos y las expectativas de mejoras podrían enriquecer las futuras directivas de manejo y control de anemia en los menores de tres años.

## MATERIALES Y MÉTODOS

### Diseño

El diseño del estudio fue fenomenológico ^18^, porque buscó comprender las percepciones y las experiencias del profesional de salud durante los procesos que se dieron al implementar la norma de manejo y control de la anemia.

### Selección de los participantes

Los participantes del estudio fueron los profesionales de la salud, médicos, enfermeras y nutricionistas que trabajaban en servicios de salud del primer nivel de atención del MINSA en Lima Metropolitana, y estaban encargados del programa de suplementación con hierro a niños menores de tres años según la Directiva Sanitaria 099-2020 ^15^. El criterio de elegibilidad fue que estuvieran laborando en forma presencial en su servicio; 33 de 51 profesionales invitados aceptaron participar voluntariamente en las entrevistas semiestructuradas a profundidad. Los que no aceptaron participar fue por no contar con disponibilidad de tiempo.

Los profesionales de la salud que participaron fueron invitados mediante llamadas telefónicas para colaborar con una entrevista semiestructurada virtual. El muestreo fue por conveniencia e intencionado hasta lograr la saturación de la información ^19^. Es decir, ya no se encontraron nuevos elementos importantes en torno al objetivo del estudio y todos los investigadores consideraron que el número de entrevistas realizadas suministraban suficiente información.

### Recolección de datos

Se aplicó una entrevista semiestructurada a profundidad ^20^ en la hora acordada con el profesional. Los profesionales de la salud trabajaban en diferentes servicios del primer nivel de atención madre-niño, tales como medicina, enfermería y nutrición; todos manejaban las directrices sobre el control de la anemia. Las entrevistas se llevaron a cabo entre los meses de noviembre del 2020 a enero del 2021; la duración fue aproximadamente de 53 min. Todas las entrevistas fueron realizadas de forma virtual mediante el aplicativo Zoom.us y conducidas por un nutricionista con experiencia en el recojo de datos cualitativos. Cabe precisar que los investigadores no laboraban en ningún establecimiento de salud de primer nivel del MINSA y no tenían vínculo laboral con los entrevistados. Todas las entrevistas fueron grabadas para incrementar la atención durante la entrevista y facilitar el análisis posterior. Se elaboró una guía semiestructurada que contenía preguntas abiertas sobre los siguientes temas: factibilidad de aplicación de la norma, percepción y experiencias sobre la suficiencia de la norma, aspectos menos factibles de cumplir y cambios normativos en el periodo 2017-2020. La guía semiestructurada tuvo la finalidad de garantizar que se cubrieran todos los temas, sirvió de patrón para la entrevista y fue probada previamente con una población con las mismas características.

### Análisis de datos

Todas las entrevistas grabadas fueron transcritas literalmente en formato Word. Los textos transcritos se sometieron a un análisis temático ^21^. El primer paso del análisis fue leer todos los textos varias veces para tener una comprensión general de los datos por todos los investigadores. Seguidamente, se organizaron los archivos en el software NVivo (QSR International, versión Release 1.5.1-2020) ^22^ el proceso de codificación fue bastante complejo debido a la dimensión de las preguntas y por el análisis de los investigadores. Las codificaciones fueron realizadas por los tres primeros autores (DDP, SE y JPA) en forma independiente, se generaron códigos de cada unidad de análisis que luego fue comparado entre investigadores. Se crearon códigos iniciales de acuerdo con un libro de códigos previamente organizado como base para el análisis. A continuación, se buscaron categorías significativas de cada tema y se creó un libro de códigos emergente (material suplementario, anexo 1), las cuales se agruparon en subtemas y temas. Para el proceso de análisis se aplicó una triangulación de investigadores ^23^. Después, los hallazgos se compararon en varias reuniones de trabajo para una mejor comprensión de los datos. Además, los resultados se discutieron, interpretaron y consensuaron entre los cuatro investigadores para incrementar la credibilidad. El nivel de consenso entre diferentes investigadores de la misma realidad eleva la credibilidad, así como la seguridad de que el nivel de congruencia de los fenómenos en estudio sea fuerte y sólido ^24^. Se completó la lista de evaluación COREQ ^25^ ^) ^de 32 ítems (material suplementario, anexo 2).

### Aspectos éticos

El estudio fue aprobado por el Comité de ética en Investigación de la Facultad de Medicina de la Universidad Nacional Mayor de San Marcos (30-10-2020). El estudio fue registrado en la plataforma PRISA con registro EI00002115.

## RESULTADOS

Se entrevistaron a 33 profesionales de la salud, 4 médicos, 6 enfermeras y 23 nutricionistas, con edad promedio de 39,8 (DE: 10,5) años. El 80% fue del sexo femenino (material suplementario, anexo 3). Se identificaron cuatro temas sobre la norma: factibilidad de aplicación, imperfecciones percibidas, desafíos en la aplicación y perspectivas de mejora para el manejo y control de la anemia (Figura 1).

**Figura 1:** Temas y subtemas sobre percepciones y experiencias de los profesionales de la salud sobre la norma del manejo y control de la anemia en menores de tres años, durante la pandemia de la COVID-19.

### Tema 1. Factibilidad de aplicación

*Valoración de aplicación de la norma*

Veintiséis participantes reconocieron la necesidad de actualizar la norma; al respecto mencionaron que al iniciar la pandemia había mucha incertidumbre de cómo organizar las atenciones de salud, si bien existían varias propuestas, no había una oficial; este vacío se llenó en parte con la norma «Directiva Sanitaria N.° 99». Asimismo, seis de ellos manifestaron que la normativa facilitó el trabajo en el manejo preventivo y tratamiento de la anemia al ser esta específica y detallada, lo que permitió su aplicación (Tabla 1).

**Tabla 1:** Percepciones sobre la factibilidad de aplicar la norma sobre manejo y control de la anemia en menores tres años.

Temas/categorías	Citas
Tema 1. Factibilidad de aplicar la norma	
Valoración de la aplicación de la norma	
Importancia de la actualización de la norma	*Umm, bueno, siempre le adicionan un poco más de alternativas según las circunstancias, por ejemplo, la última (la directiva sanitaria 099) ya nos habla del tema del COVID y cómo podemos manejar (la prevención y tratamiento de la anemia); la anterior creo que es un poco más general, nos hablaba de todos los grupos etarios* **Participante 12, nutricionista, edad 32 años**
Continuidad del manejo anemia	*…No había una orientación de cómo hacer este trabajo fundamental en el país ¿no? de combatir la anemia que está muy arraigada en los niños menores de tres años y entonces este… nos ayuda bastante para tratar de manejarlo desde casa, manejarlo con teleorientación, telemonitoreo...* **Participante 28, médico cirujano, edad 62 años**
Realidades que se enfrenta ante la aplicación	
Complejidad en la implementación de la nueva norma	*Ha sido de verdad un trabajo arduo, teníamos que estar en reuniones, tras reuniones, empaparnos bien de la norma, coordinar con los responsables de cada área, porque era una coordinación no solamente en mi caso con la médico, sino tenemos que coordinar también con la técnica, con la enfermera, con la obstetra, entonces teníamos que hacer una coordinación de tal manera que no expongamos a la población y no exponernos nosotros…* **Participante 2, nutricionista, edad 31 años**
Escaso recurso informático	*Por ejemplo; yo he tenido que tener (sic) otro celular para hacer las llamadas porque llamar de mi propio celular…, a veces a cualquier hora me mensajeaban, sábados y domingos, no había hora; ese, por ejemplo, era un problema con los pacientes COVID o de anemia te escriben a cualquier hora del día como si fuéramos call center.* **Participante 25 nutricionista, edad 36 años** *Sigue habiendo muchas aristas, porque para poder realizar una buena teleorientación y un buen telemonitoreo necesitamos también de herramientas digitales que vayan a la par con esto, o sea, sí, esto nos fortalece porque es un mundo diferente…* **Participante 26, nutricionista, edad 31 años**
Manejo de la anemia	
Desabastecimiento de hierro polimaltosado	*En sí, no es la norma lo que cambia, son los anexos. Por ejemplo, si te das cuenta en la suplementación de hierro, solamente se mencionan gotas… te habla de hierro polimaltosado, sulfato ferrosos y micronutrientes, está desactualizado; cuando actualmente no hay ni hierro polimaltosado, ya no hay ni gotas de sulfato ferroso, y micronutrientes mucho menos desde hace tiempo…* **Participante 11, nutricionista, edad 34 años**
Manejo de efectos adversos	*Bueno yo creo que en la norma debería ser más específica en cómo trata los efectos adversos que produce el suplemento, el hierro y cómo hacer si el niño no continua con la suplementación* **Participante 22, enfermera, edad 28 años**
Indicaciones de cuando se interrumpe la suplementación	*… Sí sería necesario que la norma sea explícita en el caso de los niños que han interrumpido su tratamiento por mucho tiempo o más de 6 meses, porque muchos de ellos no han venido desde marzo, han llegado recién en julio o en septiembre y han pasado 6 meses desde la última visita o desde…el último control del tratamiento anemia.* **Participante 2, nutricionista, edad 37 años**
Medición de hemoglobina como seguimiento	*El otro punto que no especifica es el dosaje de hemoglobina, porque antes se trabajaba en el caso de niños con anemia, nos indicaba al mes, a los tres meses y a los 6 meses; pero ahora es al diagnóstico, y de ahí al término del tratamiento, pero están las otras normativas pendientes y, entre comillas, se asumen, porque si ven que no se exponga al niño cuando viene, pero cuando viene nuevamente por alguna vacuna, viene el detalle de que se le tomó o no se le tomó. Por motivo de seguimiento se le ha tomado, pero en la normativa no lo define como tal.* **Participante 3, nutricionista, edad 35 años**

Fuente: elaboración propia

Además, dieciséis de ellos expresaron sus experiencias y, gracias a la norma que incluye estrategias como las citas virtuales, la teleorientación y el telemonitoreo, lograron continuar con la suplementación preventiva y el tratamiento (Tabla 1).

*Realidades que enfrentar ante la implementación*

Más de la mitad de los participantes expresaron que emocionalmente no estaban preparados para continuar con su trabajo debido al temor al contagio de la COVID-19 y por no saber cómo implementar y cumplir con las atenciones en estas circunstancias, por lo que experimentaron incertidumbre. Además, mencionaron que trabajaron arduamente, primero en conocer la nueva normativa y después en planear e implementar las nuevas estrategias, especialmente en la flexibilidad del tiempo de entrega del suplemento, un nuevo flujo de atención, ambientes adaptados, etc. Fue necesario un trabajo coordinado y la disposición de todo el equipo multidisciplinario (Tabla 1).

La norma incluía modificaciones en procedimientos habituales de atención, especialmente en el monitoreo de las consejerías en forma virtual. Muchos manifestaron haber experimentado dificultades para aplicarlo, una de ellas fue por ausencia y/o limitados recursos tecnológicos, tales como no contar con una computadora, o no tener crédito para hacer llamadas a los pacientes. Doce de ellos expresaron su disconformidad y malestar por la falta de estos equipos básicos y tuvieron que utilizar sus teléfonos personales para hacer las llamadas telefónicas. Esto les generó recibir llamadas telefónicas fuera del horario de trabajo e incluso sábado y domingo, a veces las llamadas eran para otro tipo de atenciones (Tabla 1).

*Manejo de la anemia*

Una de las complicaciones del tratamiento de la anemia fue el manejo de los efectos adversos. La gran mayoría manifestó que hubo desabastecimiento del suplemento polimaltosado, que presenta menos efectos adversos. Veintiún participantes informaron que debe incluirse en la norma cómo tratar los efectos adversos que produce el suplemento de hierro (Tabla 1). Asimismo, diez participantes manifestaron que hubo dificultad en el registro de niños con tratamiento interrumpido y consideraron que debe estar explícito el procedimiento para continuar con la suplementación con hierro cuando se interrumpe por un tiempo mayor a seis meses. Esta situación se presentó con mucha frecuencia durante la pandemia (Tabla 1).

### Tema 2. Imperfecciones percibidas

*Vacíos normativos*

La pandemia de la COVID-19 desplazó a la población, ocasionando mayor movilidad y cambios de domicilio. Este hecho estuvo relacionado con la falta de continuidad del tratamiento de la anemia. Los participantes expresaron vacíos normativos, dieciséis de ellos declararon sobre las situaciones especiales que encontraron al momento de la atención de la suplementación. Algunos de los profesionales entrevistados percibieron que la norma no precisaba condiciones especiales de suplementación, como pacientes que antes no llegaban a atenderse en el primer nivel de atención, pero por la situación de la pandemia ahora sí acudían, por ejemplo: niños prematuros o con muy bajo peso al nacer (Tabla 2). 

**Tabla 2:** Imperfecciones percibidas en la norma sobre manejo y control de la anemia en menores tres años

Temas/categorías	Citas
Tema 2. Imperfecciones percibidas en la norma	
Vacíos normativos	
Situaciones especiales para la dosificación	*Bueno, establece que niño con muy bajo peso al nacer como que lo enfocan más en centros maternos, pero ya en establecimiento del nivel 1-2, 1-3 están llegando estos pacientes y en este caso el personal médico no lo ha manejado como debe ser...* **Participante 3, nutricionista, edad 35 años**
Indicaciones sobre qué hacer con pacientes recuperados y continuadores	*En pacientes recuperados, eh…no menciona nada, tal vez con los que terminaron su TA (tratamiento de alta), los que terminaron su tratamiento, tampoco mencionan la importancia de eso o cómo se va a manejar, solo hay una codificación he visto…bueno tampoco lo dice ahí (en la norma), eso es lo que…no nos menciona mucho de eso, manejo de pacientes recuperados de anemia.* **Participante 12, nutricionista, edad 32 años**
Aspectos que deben ser más explícitos	
Cobertura de entrega según jurisdicción	*Si bien es cierto la normativa dice, tanto esta como la anterior, no discrimina tenga seguro o no tenga seguro, pero…en esta nos pide, por ejemplo, que hagamos a todos los niños, se hace a todos los niños, pero ahora por ejemplo tenemos niños que vienen de diferentes lugares y jurisdicciones …a quien no se les puede negar el dosaje de hemoglobina si es que está en un rango de niños menores de 3 años. La directiva te dice entrega suplemento tengas o no tengas, pero el problema viene cuando se hace el seguimiento...* **Participante 3, nutricionista, edad 35 años**
Incorporar al nutricionista en prescripción del suplemento	*… en la actividad preventiva en lo que es promoción de la salud, en ese aspecto interviene, pero igual...ya sea para niños de 4 meses, para niños de 6 meses sin anemia o con anemia lo entrega el médico y pone en el HIS y todo, y de verdad que para mí y no sé si desde el colegio se puede hacer esto, pero tenemos tanto o la misma capacidad de un médico creo yo en poder indicar el suplemento que no es nada del otro mundo, por el contrario pienso yo que podríamos abarcar un poco más y es una incomodidad que siempre la he tenido… como le vuelvo a repetir, la prioridad este…o el eje principal, quien da la receta es el profesional nutricionista y no es considerado dentro de la actividad* **Participante 4, nutricionista, edad 33 años**
Mayor precisión de mensajes para consejería	
Incidir en mensajes para consejería	*Yo creo que se debería procurar aumentar la incidencia en la lactancia materna exclusiva, siento que ese es un punto débil en lo que no se le está dando importancia necesaria como una actividad preventiva de anemia.* **Participante 20, nutricionista, edad 42 años**
Indicaciones sobre herramientas y material educativo de soporte	*…dar algunos tips que sean más simples para que el profesional pueda brindar a la comunidad. Un tip pequeño, corto de promoción de estilo de vida saludable...* **Participante 28, médico cirujano, edad 62 años**

Fuente: elaboración propia

Otros manifestaron que no sabían si entregar la dosis de tratamiento completo o solo para unos meses. Esto presentó inconvenientes en los registros de pacientes recuperados; así, ocho participantes mencionaron que no estaba claro el procedimiento para este registro (Tabla 2).

*Aspectos que deben ser más explícitos*

Los participantes también mencionaron que la cobertura de atención según jurisdicción debe estar claramente indicada en la norma. Esto, con el fin de mantener un orden de entrega y seguimiento de pacientes. De esa forma no habría inconvenientes en la medición de los indicadores en el manejo y control de la anemia (Tabla 2).

Por otro lado, siete de los participantes, especialmente los nutricionistas, informaron que su rol no está debidamente definido en la norma, y que deberían liderar el proceso. Si bien el nutricionista realiza actividades preventivas sobre anemia, sienten que para el tratamiento están limitados, pues no pueden prescribir el suplemento ni registrar sus atenciones (Tabla 2).

*Mayor precisión de mensajes para consejería*

Veintiún entrevistados sugirieron que se debería incorporar el término de «consejerías personalizadas» para plasmar la actividad que realmente se viene realizando. Esta consiste en absolver las interrogantes y aclarar dudas de cada madre. Asimismo, se requiere precisar la duración y el momento en que se debe brindar esta consejería para que sea oportuna (Tabla 2).

Además, reconocieron que la norma no detalla mensajes ni orientación sobre las diferentes actividades virtuales. Enfatizaron en la necesidad de contar con material educativo electrónico para difusión con mensajes prácticos, cortos y con un lenguaje sencillo (Tabla 2).

### Tema 3. Desafíos en la aplicación

*Adecuación de procedimientos*

Los participantes contaron sus experiencias durante las restricciones de movilidad. Once de ellos manifestaron que fue necesario distribuir tareas, en actividades de atención y seguimiento, entre el poco personal de salud que había en su establecimiento. Otros mencionaron que fortalecieron la forma de compartir y delegar funciones para lograr sus objetivos (Tabla 3). Doce de ellos optaron por optimizar el flujo de atención a niños con anemia para mantener un orden y cumplir con las medidas de distanciamiento social (Tabla 3).

**Tabla 3:** Desafíos al aplicar la norma sobre manejo y control de la anemia sobre manejo y control de la anemia en menores tres años.

Temas/categorías	Citas
Tema 3. Desafíos al aplicar la norma	
Adecuación de procedimientos	
Distribución de tareas	*Como hay personal que aún está haciendo trabajo remoto, se coordina con ellos para que ellos puedan llamar a estos, a estas mamás para que vengan y también trabajamos con lo que es la meta 4, las promotoras de salud, por ejemplo, a ellos le dan una relación de niños de 4 meses para que ellos puedan visitar y nosotros tenemos que trabajar con ellos y ver que niños no tienen el suplemento...* **Participante 16, nutricionista, edad 46 años**
Mejora en el flujo de atención	*Nutrición es el que se está encargando en esta primera instancia de hacer el dosaje (hay laboratorio en centro materno, licenciada) si hay laboratorio, pero en este caso eh… nosotros, tanto enfermería o nutrición está haciendo directamente ahí en consulta (el dosaje) Sí tenemos ahí nuestros equipos, los hemoglobinómetros; sí, disponemos de esos en cada área, y ahí se realizan, y ahí mismo se ve pues el resultado y de acuerdo a lo que es el resultado, bueno vuelve a pasar con enfermería para hacer la parte preventiva o con medicina.* **Participante 31, enfermera, edad 37 años**
Consideraciones de bioseguridad para ambientes pequeños	*Entonces, la solución que se ha dado es que se ha hecho otra puerta diferente para las mamás que presenta a los niños sin riesgo y otra puerta para los pacientes que tienen COVID otro camino, otra ruta que no cruza. Entonces, de esa manera se está protegiendo. Por otro lado, también se ha hecho como muritos, entonces yo creo que no hay problema …* **Participante 30, nutricionista, edad 65 años**
«Ajuste» a la norma	
Adaptación del proceso de la receta	*…Nos dieron la potestad de que pudiéramos hacer como visita, entregas (del suplemento), y que el médico después codificase (hiciera el registro HIS), pero yo ya he coordinado con el médico para yo poder realizar la receta, poder llenarla...incluso él me facilita con el tema del...del HIS, eh...llenando con su sello...(otras veces) sella (el médico) la receta, pero yo ya lo dejo hecho, y eso contribuye más a que la mamá no tenga que esperar tanto, y se pueda entregarle rápidamente y pueda eh...evitar aglomeración.* **Participante 12, nutricionista, edad 32 años**
Asumir nuevas formas de atención en salud	
Cambios en la modalidad de atención en salud	*Sí, sí coincido con esa parte de que nos abre un mundo completamente diferente al que habíamos llevado hasta hace antes del COVID ¿no?, un mundo completamente diferente de como acércanos a la mamá, y ahora no necesariamente teniéndola al frente de nosotros físicamente ¿no? facilita bastante lo que puede ser el seguimiento, si es que se sabe llevar, creo yo…* **Participante 26, nutricionista, edad 31 años**
Desacuerdo con la dosificación estandarizada	…Para mi parecer la dosis que se le ha estado dando por grupo etario es muy baja, eso no nos ayuda a fortalecer a que el niño se puede suplementar como lo hacíamos antes de acuerdo al peso. La suplementación debió ser en un promedio de lo que se manejaba… **Participante 14, enfermera, edad 35 años**

Fuente: elaboración propia

Otros se organizaron para actividades de seguimiento telefónico a la suplementación con hierro y visitas domiciliarias con equipo de bioseguridad adecuado. Siete de ellos manifestaron sus experiencias en el acondicionamiento de ambientes, a fin de evitar el contagio por la COVID-19 (Tabla 3).

*Ajuste de la norma*

La poca disponibilidad de médicos llevó a un «ajuste» o adaptación propia de los procesos de atención de la suplementación durante la inmovilización social. Once de los participantes informaron que en algunos establecimientos de salud hubo un acuerdo interno para facilitar el trabajo de la suplementación. Es decir, como parte del «ajuste» de la norma para su aplicación en la atención diaria, se realizaron acciones que se desviaban de esta directiva. Una de ellas fue la entrega del suplemento por el profesional no médico con una receta médica lista o por regularizar con la firma y sello del médico. Todo esto con la finalidad de que la madre no espere mucho tiempo (Tabla 3). Estas prácticas produjeron cierta confusión al inicio, pues por tratar de mejorar la distribución de suplementos, realizaban acciones fuera de la norma. 

*Asumir nuevas formas de atenciones en salud*

Doce de los entrevistados reconocieron que los cambios indicados en la norma respecto a consultas o seguimiento del estado de salud, les obligó a adaptarse y superar las barreras del distanciamiento social. Manifestaron que anteriormente tenían una gran resistencia a la teleconsulta, el telemonitoreo y el seguimiento telefónico. Al respecto reconocieron que para asumir estos nuevos retos son muy importantes las Tecnologías de Información y Comunicación (TIC) y que requieren preparación, por lo que solicitaron ser capacitados para facilitar su trabajo y realizarlo adecuadamente. Otros aprovecharon el uso de aplicativos conocidos por ellos y contactaron a las madres vía telefónica, lo que abrió más posibilidades de orientación para algunos, mientras que algunos temen que la madre se canse de recibir llamadas continuas (Tabla 3).

Otra práctica que tuvieron que acoger de la Directiva Sanitaria 099-2020 fue la dosis estandarizada de suplementos de hierro según edad. Dieciséis profesionales de la salud estaban de acuerdo con esta práctica, porque reconocían que, durante el contexto más crítico de la pandemia, era poco factible tomar el peso del niño; sobre todo porque se expondrían junto con la madre al contagio de la COVID-19. Sin embargo, once participantes manifestaron directamente su desacuerdo con la estandarización de las dosis por peso (Tabla 3).

### Tema 4. Perspectivas de mejora

*Implementar acciones para mejorar el desempeño*

Frente a las dificultades los participantes mencionaron acciones que se podrían mejorar en la normativa, como la frecuencia semanal del seguimiento virtual. Veinte de los participantes manifestaron que el seguimiento telefónico fue difícil por diferentes razones. Uno de ellos fue el personal insuficiente y consideraron que se deberían establecer metas más realistas a las posibilidades de los establecimientos de salud (Tabla 4).

**Tabla 4:** Perspectivas de mejora de la norma sobre manejo y control de la anemia en menores tres años.

Temas/categorías	Citas
Tema 4. Perspectivas de mejora	
Implementar acciones para mejorar desempeño	
Sincerar seguimiento	*…difícil de cumplir, lo que específica con respecto al seguimiento telefónico, sí es un buen apoyo, pero no lo podríamos hacer únicamente nosotros, ahí se podría incluir que sea más claro con respecto a que el seguimiento lo podría hacer cualquier personal técnico o administrativo… Esa sería una fortaleza, la indicación del seguimiento.* **Participante 2, nutricionista, edad 37años**
Esclarecer pautas de seguimiento	*…creo que tiene pautas que llevar cada uno, cómo realizarlas y los compromisos al final, también cómo deben ser esos compromisos, cuántos, porque tampoco se le va a dar toda la información por medio telefónico, porque no te va a captar, o sea, yo creo que faltaría especificar cómo debería ser este seguimiento telefónico…* **Participante 31, enfermera, edad 37años**
Implementar acciones de mejorar el desempeño	*…nos dicen a realizar la teleorientación, pero no nos dan unos, no sé, los, las cómo decirlo, las estrategias para poder llevar a cabo estas teleorientaciones, también es un tema poder nosotros incluir estas actividades que normalmente nosotros hacíamos, la consulta en el consultorio no teníamos que llenar nada y para nosotros, inclusive para las personas mayores ha sido un poco más tedioso poder realizar estas teleorientaciones a través de las nuevas plataformas.* **Participante 27, nutricionista, edad 31años**
Funcionalidad de la norma	
Todo niño debe pasar por servicio nutrición	*…el tema de la parte de nutrición…la parte de la consulta de nutrición de repente…porque esa es parte importante, no dice (la norma) que el niño debe ir con nutrición, no dice nada de eso, solamente dice que hay mensajes claves (que debe recibir) que tiene que manejar eso, pero la gran mayoría de los centros tiene nutricionista.* **Participante 12, nutricionista, edad 32años**
Equipo atención exclusivo	*… nos piden que atendamos a todos y… u otra forma de repente ya si no hay plata para un equipo exclusivo, otra estrategia para atender de manera más masiva... que debe haber un nutricionista para niños y un nutricionista para adultos, procurar hacer el flujo más corto, procurar también que… o que hay un equipo exclusivo para que dejen que el nutricionista cumpla su función de nutricionista en realidad. Y deje de estar haciendo las funciones administrativas.* **Participante 32, nutricionista, edad 41años**
Revisar instrumento de medición de vulnerabilidad	Un día, de repente, no sé…ese día pasó algo, se enfermó el niño, de repente no pudo comer por algún motivo los tres grupos de alimentos, y de repente, y no quiere decir que todos los días coma así ¿no? De repente sería un registro más…o un análisis en el monitoreo más profundo, o ver en la semana o en el mes, pero no regirse específicamente a un día, que es el día anterior, que no me parece exacto para contemplar a alguien vulnerable o clasificarlo como vulnerable ¿no? **Participante 7, nutricionista, edad 28años**

Fuente: elaboración propia

Once participantes consideraron que la madre debe comprometerse con el cumplimiento del tratamiento. También manifestaron que debe especificarse cómo supervisar el seguimiento remoto, especialmente cuando no hay una plataforma virtual del establecimiento de salud (Tabla 4).

Otro aspecto que recomendaron los participantes fue que el MINSA debería capacitar en el manejo y control de la anemia a todo el personal de salud, incluyendo a los técnicos. Entre las acciones de capacitación deben incluir experiencias exitosas en el telemonitoreo y teleconsulta de otros establecimientos. Por otro lado, manifestaron que fue muy tedioso el uso de plataformas virtuales, y consideraron que debería haber más asesoría virtual (Tabla 4).

*Funcionalidad de la norma*

Los participantes manifestaron que la norma debe ser más funcional (más práctica y útil); entre las recomendaciones estuvo tener un sistema automatizado, y ampliar las indicaciones sobre la medición de la hemoglobina. Doce de ellos mencionaron que se debe especificar que todo niño atendido debe pasar por el servicio de nutrición, siempre y cuando el establecimiento cuente con un nutricionista. Además, tener un equipo de atención exclusivo para la anemia (Tabla 4).

## DISCUSIÓN

Este estudio brinda una visión general sobre las percepciones y experiencias del profesional de salud acerca de la norma vigente para el manejo y tratamiento de anemia en niños menores de tres años durante la pandemia de la COVID-19 en el año 2020. En este sentido, se encontró que el profesional de salud percibió que la norma era útil y factible de aplicar; además, expresaron sus experiencias vividas en la aplicación del manejo y control de la anemia con pocos recursos y nuevas disposiciones de atención como el uso de medios virtuales. Sin embargo, muchos encontraron desafíos en la ejecución y, en algunos casos, adecuaron procedimientos para la atención de madres y niños. Asimismo, la responsabilidad de atender a los niños en un contexto de emergencia, el temor al contagio y los cambios tan repentinos, fueron retos asumidos por el profesional de la salud y, en el marco de esa experiencia, manifestaron sus perspectivas de mejora a la normativa para cumplir con el manejo de la anemia.

Según los resultados, varios de los profesionales de la salud manifestaron la factibilidad de aplicar la norma en contexto con la pandemia de la COVID-19. Algunos consideraron que era una actualización de la norma anterior o un anexo, pero que contribuyó al retorno a sus actividades preventivas y recuperativas de la anemia. Según los relatos de los médicos, enfermeras y nutricionistas uno de los aspectos más importantes fue que incluyeron atenciones virtuales tales como la teleorientación y seguimiento telefónico. Esto les permitió superar la distancia y continuar con la suplementación. Un estudio en profesionales de la salud encontró que la comunicación telefónica facilitó el seguimiento al paciente, especialmente para gestionar las citas ^26^, similar a lo encontrado en nuestro estudio, aunque hubo una recarga de las actividades del personal, especialmente para hacer el seguimiento al paciente. Además, trabajos previos muestran que la comunicación entre el proveedor del servicio y el paciente mejora el éxito del tratamiento y adherencia al tratamiento^ (^ ^27^. Por otro lado, las estrategias implementadas como distribución de tareas y mejora del flujo de atención permitieron adaptarse al contexto y mejorar las atenciones.

En muchos países se elaboran directrices nacionales para el tratamiento de una enfermedad, un caso en particular es la anemia en menores de tres años. Sin embargo, existen varios desafíos que están involucrados en su implementación. En el presente estudio, al igual que el de Helseth* et al.* ^28^, al personal de salud se les asignaron nuevas tareas y responsabilidades con la nueva directiva, pero estos sentían que no estaban suficientemente preparados, ni tampoco contaban con el apoyo de recursos adicionales. Los profesionales de la salud identificaron desafíos en la implementación de las directrices en varios niveles: a nivel del sistema de salud (recursos del establecimiento de salud); nivel del personal de salud (competencia percibida, carga de responsabilidad, actitudes); nivel del tratamiento (dosificación de suplementos e instrumentos de monitoreo), y a nivel profesional (escepticismo ante la dosis estandarizada de suplementos de hierro). Todo ello repercutió en las bajas expectativas de los profesionales de salud frente a la norma. Otros estudios muestran, además de los inconvenientes mencionados, la proliferación de guías, la redundancia de los temas abordados y sesgos en su metodología^ (^ ^29^.

Debido a la urgente implementación de la normativa frente al contexto de la pandemia, se tuvieron que adaptar muchos procedimientos. Si bien una guía es prioritaria para la toma de decisiones en la atención médica, su aceptabilidad y uso por parte del profesional de salud es compleja y está influenciada por varios factores como la profesión, el conocimiento, las percepciones y sus actitudes. Según Solá *et al*., el uso de las guías en la práctica diaria implica un proceso de implementación, que involucra la difusión y capacitación en estos documentos, elementos facilitadores que intervienen en un proceso de retroalimentación positiva^ (^ ^29^.

En el estudio se muestran actividades y procedimientos que se han ajustado para cumplir con la directiva, evidenciando que habría aspectos por revisar. A falta de médicos, varios de los profesionales entrevistados (enfermeras y nutricionistas) tuvieron que optar por la entrega del suplemento y después completar el procedimiento de la receta médica. Además, tuvieron que distribuir las tareas entre el profesional disponible para el manejo de la anemia, mejorar el flujo de atención presencial y cambios en la modalidad de brindar consultas y seguimiento del estado de salud. Ello nos indica que la actual directiva adolece de precisiones y requiere adaptarse a la realidad de cada establecimiento y región, además de tener mecanismos para regularlos. La identificación de barreras para la implementación y el cumplimiento de las directrices deben analizarse con anticipación para que se puedan desarrollar estrategias que se adapten al entorno específico.

La pandemia de la COVID-19 se impuso en el mundo interrumpiendo todas las actividades humanas, principalmente por el temor al contagio del SARS-CoV-2. En este contexto, las atenciones para el tratamiento de anemia descendieron abruptamente. Frente a esta realidad surgió la Directiva Sanitaria 099-2020, como una respuesta de adecuación para garantizar la continuidad de las acciones contra la anemia ^15^; sin embargo la rapidez con que apareció la COVID-19, los grandes cambios económicos, sociales, sanitarios, psicológicos y las nuevas situaciones en la atención de salud hicieron difícil que una norma fuera suficiente para abordar este escenario complejo y dinámico de la pandemia.

Esta insuficiencia de la norma fue percibida por el profesional de salud que reveló vacíos, enfatizando que no se incluyeron indicaciones para la atención de pacientes que no acudían a establecimientos de primer nivel como niños prematuros, de bajo peso al nacer, además niños migrantes que proceden de zonas de altitud; también mencionaron que no hay indicaciones para un seguimiento continuo de niños en condiciones de movilidad (migrantes). Estos vacíos de las normas durante la pandemia también han sido reportados en otros estudios como el de Shahil Feroz y Pradhan en Pakistán, donde el personal de salud tuvo que proveer atención médica sin procedimientos claros ^30^, y el estudio de Iyer *et al*. realizado en la India, que recomienda elaborar guías de práctica (en este caso quirúrgicas) adecuadas a la pandemia, además de actualizarlas periódicamente ^31^.

Otro aspecto que surge como vacío de esta norma es que no hace referencia a los medios e insumos para desarrollar la atención de salud a distancia; los entrevistados manifestaron falta de insumos, desde aspectos tan básicos como el financiamiento de las llamadas telefónicas, hasta la necesidad de una plataforma virtual y material digital para desarrollar la teleorientación y telemonitoreo. Al respecto, la evidencia muestra un impacto significativo de la telesalud para prevenir, diagnosticar y dar tratamiento en algunas enfermedades; sin embargo, la implementación de la telesalud supone algunos requisitos mínimos tanto para el prestador de salud como para el paciente y cuando no se garantizan constituyen barreras de acceso al servicio de salud^ (^ ^32^.

Las limitaciones del estudio fueron que la recolección de datos se realizó por videollamadas a través de la plataforma Zoom, lo que pudo restringir la interacción; sin embargo, dado el contexto, otros estudios también han usado esta estrategia y brindan recomendaciones, que consideramos en el estudio para optimizar el proceso ^33^. Otras limitaciones fueron que se aplicó solo una técnica de recolección de datos: entrevista semiestructurada a profundidad; sin embargo la aplicación de la entrevista de forma directa y abierta fue suficiente para saturar la información, y por último, no se devolvieron los datos a los participantes para corroborarlos. Las fortalezas del estudio fueron que para garantizar la credibilidad de los datos el estudio aplicó dos tipos de triangulación: triangulación por investigadores y triangulación de fuentes de datos, considerando los puntos de vista del profesional de salud involucrado en el tema de anemia: médicos, enfermeras y nutricionistas; además, otra fortaleza fue que la muestra estuvo constituida por profesionales de salud provenientes de todas las direcciones de salud (DIRIS) de Lima Metropolitana y del Callao.

En conclusión, los hallazgos del estudio muestran que el profesional de la salud percibió que las normas contribuyeron a continuar con el manejo de la anemia en un contexto de pandemia, y las experiencias mostraron los desafíos enfrentados por las dificultades en su aplicación, las que trataron de solucionar modificando algunas indicaciones según su realidad con el fin de lograr que los niños continúen con las atenciones para el manejo y control de la anemia. Es conveniente tomar en cuenta los aspectos positivos que manifestaron los entrevistados, como también considerar los aspectos que faltan aclarar y agregar a las futuras normas para que sean más explícitas y útiles en la lucha contra la anemia.
